# N/O Dual‐Doped Environment‐Friendly Hard Carbon as Advanced Anode for Potassium‐Ion Batteries

**DOI:** 10.1002/advs.201902547

**Published:** 2020-01-09

**Authors:** Rong Chao Cui, Bo Xu, Hou Ji Dong, Chun Cheng Yang, Qing Jiang

**Affiliations:** ^1^ Key Laboratory of Automobile Materials (Jilin University) Ministry of Education, and School of Materials Science and Engineering Jilin University Changchun 130022 China

**Keywords:** hard carbon, low cost, N/O dual doping, potassium‐ion batteries, sorghum stalks

## Abstract

Potassium‐ion batteries (PIBs) are considered as promising candidates for lithium‐ion batteries due to the abundant reserve and lower cost of K resources. However, K^+^ exhibits a larger radius than that of Li^+^, which may impede the intercalation of K^+^ into the electrode, thus resulting in poor cycling stability of PIBs. Here, an N/O dual‐doped hard carbon (NOHC) is constructed by carbonizing the renewable piths of sorghum stalks. As a PIB anode, NOHC presents a high reversible capacity (304.6 mAh g^−1^ at 0.1 A g^−1^ after 100 cycles) and superior cycling stability (189.5 mAh g^−1^ at 1 A g^−1^ after 5000 cycles). The impressive electrochemical performances can be ascribed to the super‐stable porous structure, expanded interlayer space, and N/O dual‐doping. More importantly, the NOHC can be prepared in large scale in a concise way, showing great potential for commercialization applications. This work may impel the development of low‐cost and sustainable carbon‐based materials for PIBs and other advanced energy storage devices.

## Graphical abstract

A low‐cost and sustainable N/O dual‐doped hard carbon (NOHC) is constructed by carbonizing the renewable piths of sorghum stalks. As an anode material in potassium‐ion batteries, the NOHC exhibits a high reversible capacity and superior cycling stability, outperforming most carbonaceous materials.

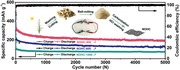

## 1 Introduction

Lithium‐ion batteries (LIBs) are the most common energy storage systems, which have been widely used in portable electric energy storage devices and electric vehicles due to their high energy and power densities.^1^, ^2^, ^3^ However, the geographical limitations and rising cost of lithium resources have greatly hindered large‐scale applications of LIBs. A series of energy storage devices has received great attentions as promising alternatives to LIBs, such as magnesium‐ion batteries, calcium‐ion batteries, aluminium‐ion batteries, sodium‐ion batteries, potassium‐ion batteries (PIBs), etc.^4^, ^5^, ^6^, ^7^, ^8^, ^9^, ^10^, ^11^, ^12^, ^13^, ^14^ Among them, PIBs represent the most attractive one owing to i) the abundant reserve and lower cost of K resources; and ii) the appropriate redox potential of K^+^/K (−2.93 V vs standard hydrogen electrode (SHE)), very close to that of Li^+^/Li (−3.04 V vs SHE), implying high voltage platform and energy density of PIBs.^15^, ^16^, ^17^, ^18^, ^19^, ^20^, ^21^ However, K^+^ exhibits a larger radius (1.38 Å) than that of Li^+^ (0.76 Å), which may impede the intercalation of K^+^ into electrodes, thus resulting in poor cycling stability and lower capacity of PIBs.^15^, ^22^ Hence, it is significant to achieve favorable structural stability of PIBs anode materials to solve this problem.

Up to now, tremendous efforts have been dedicated to developing anode materials for PIBs, such as carbonaceous materials, Mxene, metal‐based materials, phosphides, selenides, and sulfides.^13^, ^14^, ^15^, ^16^, ^17^, ^18^, ^19^, ^20^, ^21^, ^22^, ^23^, ^24^, ^25^, ^26^, ^27^, ^28^, ^29^, ^30^ Thereinto, carbon‐based materials are the extremely attractive ones due to their high electrical conductivity and chemical stability. Furthermore, they can easily withstand volume expansion and accommodate more K^+^ due to expanded interlayer spacing. It has been demonstrated that the introduction of heteroatoms (N, O, P, S, F, etc.) is an effective approach to adjust interlayer distance.^17^ Moreover, heteroatoms doping (especially multi‐component doping) or micro/mesopores could generate abundant defects, significantly increasing electrochemical active sites and thus enhancing the capacity of carbon materials.^13^, ^16^, ^17^, ^28^ For example, Ruan et al. reported a N/O dual‐doped carbon network for PIBs, which exhibits a capacity of 260 mAh g^−1^ at 0.1 A g^−1^ after 100 cycles and a capacity of 160 mAh g^−1^ after 4000 cycles at 1 A g^−1^.^17^ He et al. designed a P/N co‐doped 3D porous carbon, which displays ultrahigh reversible capacities of 419.3 and 270.4 mAh g^−1^ at 0.1 and 1 A g^−1^, respectively.^28^ However, these materials are synthesized in small‐scale and complex processes, which have prevented their potential industrial applications. Thus, it is crucial to design a concise, low‐cost, large‐scale, and environment‐friendly strategy to prepare carbonaceous materials with high reversible capacity and superior cycling stability to accelerate the commercialization of PIBs.

As promising carbonaceous materials, biomass resources are low cost, abundant, and moreover renewable. Thereinto, as the fifth most widely cultivated crops in the world,^31^ sorghum accounts for a great proportion of biomass, which creates about billions tons wastes of sorghum stalks (SSs) per year. To our knowledge, the traditional treatment of SSs through incinerating will lead to severe environmental issues. Therefore, utilizing SSs or other biomass as precursors to develop electrode materials draws more and more attentions recently.^32^, ^33^, ^34^, ^35^ SS is made of rinds and piths, and both of them consist of cellulose, lignin, and hemicellulose.^31^ Nevertheless, compared to rinds, it is more easy to prepare porous carbon materials by using piths thanks to the soft and loose structure.^31^ Moreover, SSs contain large amounts of oxygen and traces of nitrogen.^36^ The oxygen‐rich functional groups can not only generate more electrochemical active sites but also enhance the surface wettability.^35^ A certain amount of nitrogen can afford more electrochemical active sites and promote the conductivity by increasing the ion/electron diffusion rate.^28^

Taking into account the factors above, as a proof of concept, we construct a hybrid of N/O dual‐doped hard carbons (NOHCs), which are fabricated by carbonizing piths of SSs in a large scale. The obtained NOHCs present functional groups, hierarchical micro/mesopores structures, and abundant active sites. As an anode material for PIBs, NOHCs exhibit a high reversible specific capacity of 304.6 mAh g^−1^ at a current density of 0.1 A g^−1^ over 100 cycles and prominent cycling stability with a capacity of 189.5 mAh g^−1^ at 1 A g^−1^ over 5000 cycles, outperforming most carbonaceous materials.^13^, ^15^, ^16^, ^17^, ^37^, ^38^, ^39^, ^40^

## 2 Results and Discussion

### 2.1 Material Synthesis and Characterization

**Figure 1:**
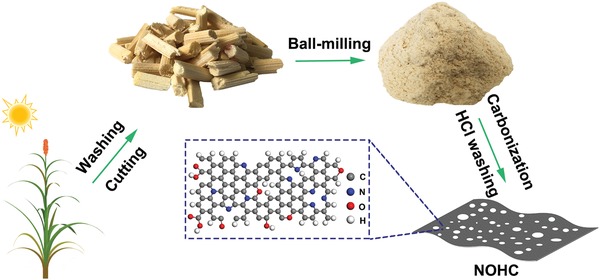
Schematic illustration of the preparation of NOHC. Heteroatoms doping (especially multi‐component doping) or micro/mesopores could generate abundant defects, significantly increasing electrochemical active sites and thus enhancing the capacity of carbon materials. Here, we construct a hybrid of porous NOHCs by carbonizing piths of SSs in a large scale, which contain large amounts of oxygen and traces of nitrogen.

The NOHCs were prepared through a facile carbonization process, which is schematically illustrated in **Figure** **1**. First, the piths of SS were cut into small pieces. Then, ball‐milling was applied to grind the piths. Finally, the NOHCs were obtained by carbonizing the ground piths at 800 °C for 2 h in Ar atmosphere, named as NOHC‐800. Note that other carbonization temperatures of 600 and 1000 °C (denoted as NOHC‐600 and NOHC‐1000, respectively) were also proven to optimize the carbonizing condition. All synthesis details are given in the Experimental Section.

**Figure 2:**
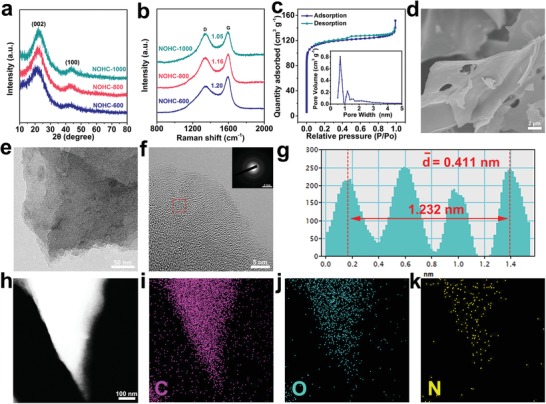
Structural morphologies of NOHCs carbonized at different temperatures. a) XRD patterns and b) Raman spectra of NOHCs. c) N_2_ adsorption–desorption isotherms of NOHC‐800. The inset shows the pore size distribution of the adsorption branch obtained by the DFT method. d–f) FESEM, TEM, and the HRTEM images of NOHC‐800. The inset in f) is a SAED image of NOHC‐800. g) Line profile is acquired from the framed area in (f). h–k) High‐angle annular dark‐field scanning TEM image of NOHC‐800 and the corresponding elemental mappings for C, O, and N elements.

X‐ray diffraction (XRD) and Raman spectroscopy were performed to explore the microstructure of NOHCs. As displayed in **Figure** **2**a, all NOHCs show two broad peaks at about 21° and 43°, which are related to (002) and (100) lattice planes, respectively, of disordered carbon structure.^32^, ^33^ As the carbonization temperature rises, NOHCs peak patterns do not change significantly, indicating the nature of hard carbon. However, the peak of (002) plane is shifted to a higher angle slightly with the temperature increasing, implying the decrease of the interlayer distance *d*_002_. According to the Bragg's law, the values of *d*_002_ are 0.419, 0.411, and 0.398 nm for NOHC‐600, NOHC‐800, and NOHC‐1000, respectively. Nevertheless, the *d*_002_ values of all NOHCs are still much higher than that of graphite (0.335 nm), which could promote the electrochemical insertion/extraction of K^+^.^37^, ^38^ For Raman spectra of all NOHCs as illustrated in Figure 2b, two separated bands are present at 1342 cm^−1^ (D band) and 1596 cm^−1^ (G band), which correspond to the disorders (or defects) and tangential vibration of carbon atoms. Moreover, the integral intensity ratio of D band to G band (*I*_D_/*I*_G_) signifies the degree of graphitic ordering.^15^ It is found that the value of *I*_D_/*I*_G_ (see **Table** **1**) decreases with increasing carbonization temperature, demonstrating an increasingly high graphitization. The N_2_ adsorption–desorption isotherms (see Figure 2c and Figure S1a,b, Supporting Information) manifest the porous nature of NOHCs. The corresponding Brunauer–Emmett–Teller (BET) surface areas of NOHC‐600, NOHC‐800, and NOHC‐1000 are 88.45, 356.98, and 566.21 m^2^ g^−1^, respectively. It is obvious that NOHCs possess large BET surface area, which increases rapidly as heat‐treatment temperature rises, suggesting the significant influence of carbonation temperature on the surface area of NOHCs. In addition, the pore size increases slightly as carbonization temperature rises, and most pores within NOHCs are smaller than 2 nm (see insets of Figure 2c and Figure S1, Supporting Information) according to the density functional theory (DFT) method. It is clear that micropores (<2 nm) account for the major part, which are generated from the reduction of functional groups, poor accumulation of C—C aromatic structures, and the increase of rotating graphene layers.^31^ The abundant micropores in NOHCs are conducive to the rapid transport of electrons and ions, while mesopores (2–3 nm) could facilitate the rapid diffusion of the electrolyte. Both of them are of great significance for K^+^ storage in PIBs.^32^, ^39^ From the field‐emission scanning electron microscope (FESEM) and transmission electron microscopy (TEM) images of NOHCs (see Figure 2d,e and Figures S2a,b and S3a,b, Supporting Information), it is observed that NOHCs exhibit lamellar structure with some big voids on the rugged surfaces. Furthermore, the loosely packed graphitic sheets within NOHCs could offer appropriate space for potassiation/depotassiation and promote the rapid ingression of the electrolyte. Additionally, the morphology shows negligible change at various temperatures. The high‐resolution TEM (HRTEM) images of NOHCs (see Figure 2f and Figure S4a,b, Supporting Information) show the disordered structure of hard carbon, manifesting the amorphous nature of the samples. All selected area electron diffraction (SAED) patterns (see the inset of Figure 2f and Figure S4a,b, Supporting Information) reveal scattered diffraction rings, further proving the amorphous nature of NOHCs. As can be seen from Figure 2g (and Figure S4c,d, Supporting Information), the average interlayer spacings of NOHC‐600, NOHC‐800, and NOHC‐1000 are 0.417, 0.411, and 0.401 nm, respectively, which are much larger than that of graphite and agree well with the XRD results. Furthermore, element mapping images demonstrate the uniform distributions of N and O over the entire flaky NOHCs (see Figure 2h–k).

**Table 1:** Structure properties and surface chemistry of NOHCs

Sample	*d*_(002)_ [nm]	*I*_D_/*I*_G_	*S*_BET_ [m^2^ g^−1^]	Element content [at%]		
				C	N	O
NOHC‐600	0.419	1.20	88.45	78.01	2.62	19.37
NOHC‐800	0.411	1.16	356.98	82.43	2.02	15.56
NOHC‐1000	0.398	1.05	566.21	90.47	1.13	8.40

**Figure 3:**
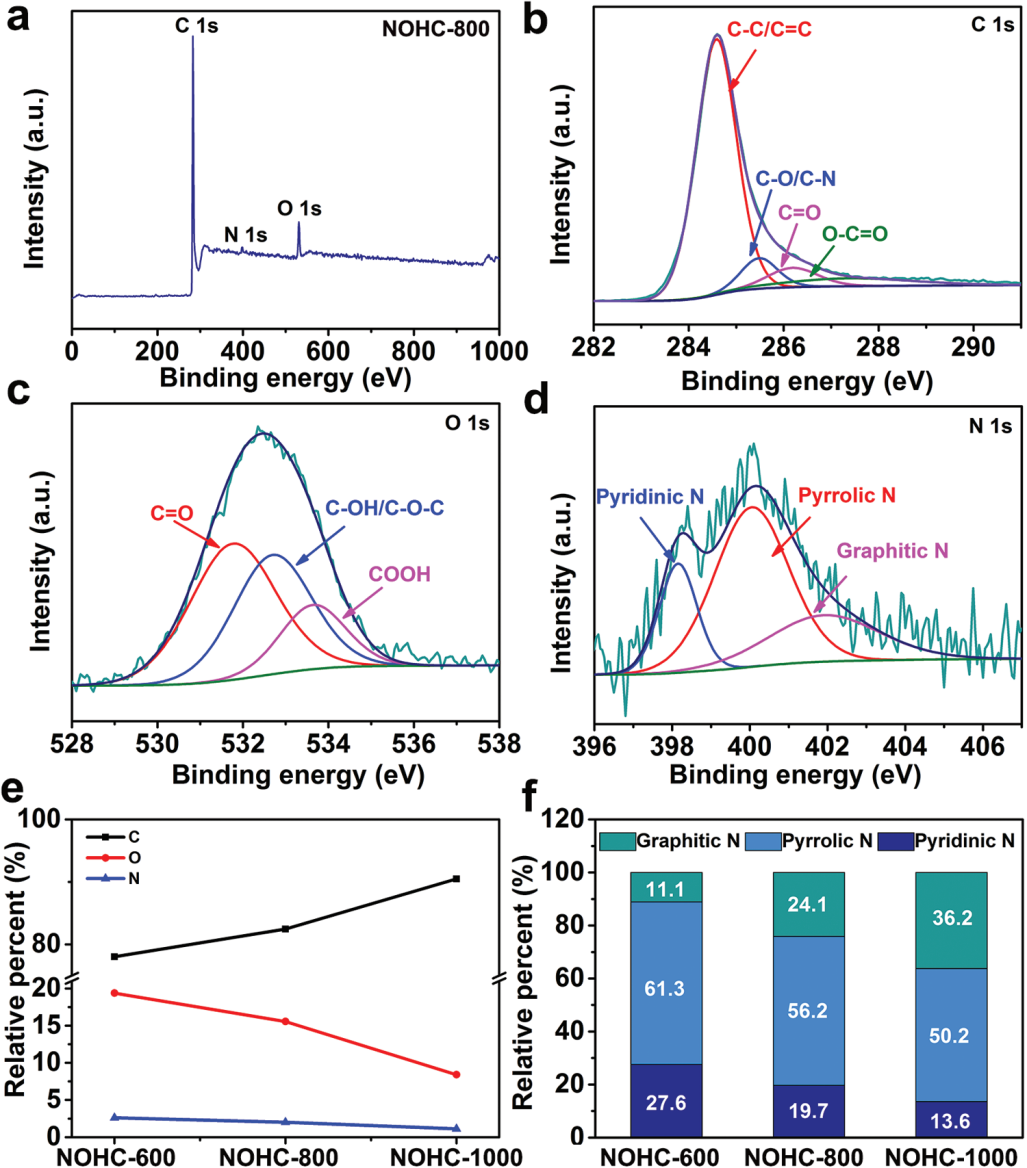
XPS spectra for NOHCs. a) The survey spectrum of NOHC‐800. b–d) The high‐resolution XPS spectra of C 1s, O 1s, and N 1s for NOHC‐800, respectively. e) The contents of C, N, and O in NOHCs. f) The contents of different species of N in NOHCs.

The X‐ray photoelectron spectroscopy (XPS) analysis was performed to investigate the elemental composition and bonding configuration of NOHCs (see **Figure** **3** and Figures S5 and S6, Supporting Information). The corresponding elemental compositions are listed in Table 1. Obviously, all NOHCs are mainly composed of C, N, and O elements, and the proportion varies at different temperatures. The high‐resolution C 1s peak of NOHC‐800 (see Figure 3b) indicates that four peaks of C—C/C=C (284.58 eV), C—O/C—N (285.48 eV), C=O (286.19 eV), and O—C=O (287.36 eV) are presented, proving in situ N/O dual‐doping in samples. As shown in Figure 3c, three typical peaks at 531.77, 532.70, and 533.64 eV are observed in O 1s spectra, corresponding to C=O, C—OH/C—O—C, and COOH groups, respectively.^32^, ^40^ Moreover, the presence of C—OH hydroxyl group could improve the surface wettability.^40^, ^41^ As a result, high specific surface area can be fully utilized and the K^+^ storage is promoted.^40^, ^41^ The N 1s XPS spectrum of NOHC‐800 (see Figure 3d) can be fitted into three peaks at 398.15, 400.05, and 401.91 eV, which are related to pyridinic N (19.7%), pyrrolic N (56.2%), and graphitic N (24.1%), respectively.^42^ A certain amount of N can afford more electrochemical active sites and enhance the conductivity by accelerating the ion/electron diffusion.^38^ Furthermore, with rising temperature, the contents of C increase in NOHCs, while the N and O contents decrease gradually (see Figure 3e). Meanwhile, the contents of pyridinic N and pyrrolic N reduce, whereas the graphitic N content increases apparently (see Figure 3f).

### 2.2 Electrochemical Properties

**Figure 4:**
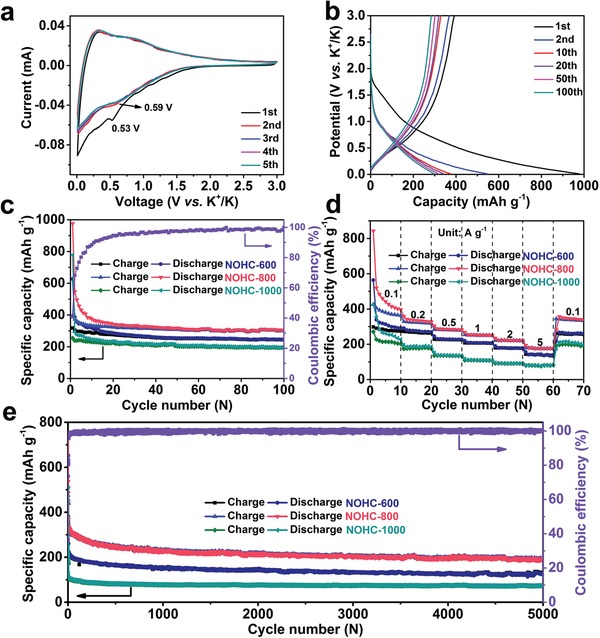
Electrochemical properties of the NOHCs electrode. a) CV curves of the NOHC‐800 electrode at a scan rate of 0.1 mV s^−1^. b) Galvanostatic charge/discharge curves of NOHC‐800 at 0.1 A g^−1^. c) Cycling performance and CE of NOHCs at 0.1 A g^−1^. d) Rate performance of NOHCs at various current densities. e) Cycling performance and CE of NOHCs at 1 A g^−1^.

To explore the K‐storage properties of the as‐prepared NOHCs, the coin‐type half‐cells were assembled by using NOHCs and K metal as the working electrode and counter electrode, respectively. A series of cyclic voltammetry (CV) analyses over NOHCs was conducted from 0.01 to 3.0 V (vs K^+^/K) with a scanning rate of 0.1 mV s^−1^ (see **Figure** **4**a and Figures S7a and S8a, Supporting Information). It is clear that the peak occurred at 0.53 V in the first cycle disappears in the subsequent cycles, which are ascribed to the formation of the solid‐electrolyte interface (SEI) layer. Meanwhile, there is always a sharp peak near the cutoff voltage in all cathodic scans, corresponding to the formation of KC_8_.^43^ Additionally, the wide anodic peak is related to the deintercalation of K^+^. During the following cycles, several broad peaks appear at around 0.69 V (NOHC‐600), 0.59 V (NOHC‐800), and 0.71 V (NOHC‐1000), which are related to the interaction of K^+^ with N atoms.^44^ Apparently, the CV curves show comparable characteristics from the second to the fifth scan, suggesting excellent reversibility of NOHCs. The galvanostatic charge and discharge curves of NOHCs are displayed in Figure 4b and Figures S7b and S8b in the Supporting Information. The initial discharge and charge capacities are 627.3 and 318.1 mAh g^−1^ for NOHC‐600, 976.4 and 398.2 mAh g^−1^ for NOHC‐800, and 778.4 and 254.6 mAh g^−1^ for NOHC‐1000 at a current density of 0.1 A g^−1^, with a corresponding initial Coulombic efficiencies (CE) of 50.7%, 40.8%, and 32.7%, respectively. Such a tendency of CE is in accordance with the specific surface area. The larger the specific surface area, the more SEI films are formed, leading to lower CE. Similar phenomena have been previously reported for carbonaceous materials in sodium‐ion batteries and PIBs.^32^, ^41^, ^44^ Apparently, NOHC‐800 has the highest reversible capacities, which originates from the best electron/ionic charge transfer kinetic characteristics according to electrochemical impedance spectroscopy (EIS) measurements (see Figure S9, Supporting Information). The capacities are high in the initial, and the subsequent loss is mainly caused by the constitution of SEI film on the NOHCs electrode surface,^15^ in consistent with the CV results. Note that NOHCs show higher reversible capacities than the theoretical capacity of graphite (279 mAh g^−1^).^15^, ^37^, ^41^ Possible reasons are: i) NOHCs possess expanded nominal interlamellar spacing in short range and thus can contribute more K^+^ during the potassiation/depotassiation process;^15^, ^45^ and ii) there are plenty of edges and defects in NOHCs owing to their amorphous nature, micro/mesopores structure, and N/O dual‐doped heteroatoms, which can provide abundant active sites and adsorb massive K^+^, resulting in the enhanced capacity ultimately.^15^ From Figure 4c, NOHC‐800 maintains a reversible capacity of 304.6 mAh g^−1^ at 0.1 A g^−1^ after 100 cycles (see Figure 4c), while NOHC‐600 and NOHC‐1000 deliver lower capacities of 245.8 and 199.1 mAh g^−1^, respectively. From the second cycle, the charge/discharge curves of all NOHCs present sloping feature with unapparent plateau, which indicates that the capacity is dominated by capacitive‐controlled process.^28^, ^44^ The rate performance of NOHCs electrodes at diverse current densities is illustrated in Figure 4d, wherein NOHC‐800 shows the best properties and its average specific capacities at 0.1, 0.2, 0.5, 1, 2, and 5 A g^−1^ are 439.1, 336.5, 286.7, 254.4, 223.4, and 178.9 mAh g^−1^, respectively, exhibiting excellent rate performance even at high current density. Additionally, the capacity of NOHC‐800 electrode is rapidly recovered and maintains stable in the following cycles when the current density returns to 0.1 A g^−1^. To evaluate the long‐term cycling durability of NOHCs electrode at high current density, the cells were tested after 5000 cycles at 1 A g^−1^ (see Figure 4e). Impressively, the NOHC‐800 electrode delivers an outstanding reversible capacity of 189.5 mAh g^−1^ after 5000 cycles, outperforming most carbonaceous anode materials for PIBs (see Table S1, Supporting Information), demonstrating its potential for practical applications. By contrast, the NOHC‐600 and NOHC‐1000 display lower reversible capacities of 125.8 and 73.5 mAh g^−1^ after 5000 cycles, respectively.

**Figure 5:**
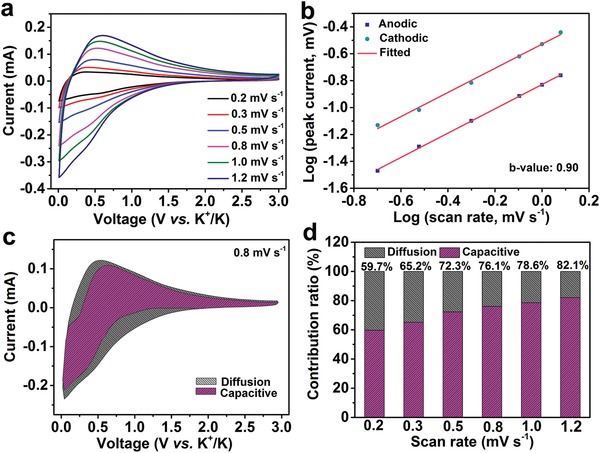
Quantitative analysis of potassium‐ion storage in NOHC‐800. a) CV curves at various scan rates from 0.2 to 1.2 mV s^−1^. b) The measurement of *b*‐value. c) Contribution of the capacitive and diffusion process at a scan rate of 0.8 mV s^−1^. d) Contribution ratios of the capacitive process at different scan rates.

To describe the kinetics and electrochemical reaction mechanism of NOHCs anode in PIBs, the CVs at different scan rates from 0.2 to 1.2 mV s^−1^ were measured, and the shape of CV curves are well maintained (see **Figure** **5**a). Generally, the current (*i*) and scan rate (*v*) can be correlated with the following power–law relationship^13^, ^14^, ^46^, ^47^, ^48^

(1) $$i=av^{b}$$

where *a* and *b* are variable constants. The *b* value can be determined by plotting log(*i*) versus log(*v*) curves. Typically, the *b*‐value of 0.5 indicates an ideal diffusion‐controlled process, while the *b*‐value of 1.0 represents a capacitive‐controlled process.^46^ In our case, both the calculated *b* values of cathodic and anodic peaks are 0.90 (see Figure 5b), indicating that the K^+^ charge storage is mainly a capacitive‐controlled process. Moreover, the specific contribution from the mixed behaviors at a fixed potential (*V*) can be differentiate from the following equation^46^, ^47^, ^48^

(2) $$i\left(V\right)=k_{1}v+k_{2}v^{1/2}$$

where *k*_1_ and *k*_2_ are fitting parameters, *k*_1_ *v* implies the capacitive‐controlled contribution and *k*_2_ *v* ^1/2^ stands for the diffusion‐controlled contribution, and their proportions can be determined by determining *k*_1_ and *k*_2_. The NOHC‐800 electrode presents a calculated capacitive charge contribution of 76.1% at 0.8 mV s^−1^ (see the shaded area in Figure 5c). As shown in Figure 5d, the capacitive contribution enhances with the increasing scan rate (from 0.2 to 1.2 mV s^−1^) and finally reaches as high as 82.1% at 1.2 mV s^−1^. Therefore, the majority of charge storage in NOHC‐800 is associated with capacitive‐controlled processes. The high proportion of capacitive behavior of the NOHC‐800 electrode should be attributed to plentiful defects arising from the intrinsic amorphous nature, micro/mesopores structure, and N/O dual‐doped heteroatoms, which could enhance the adsorption of K^+^ and thus result in better rate performance.^49^, ^50^, ^51^, ^52^, ^53^ We compared the capacitive behavior of the hard carbon electrode with a less‐defects acetylene black electrode. Figures S10–S12 in the Supporting Information show the Raman spectrum, BET surface area of acetylene black, and quantitative analysis of potassium‐ion storage in acetylene black, respectively. The acetylene black presents lower *I*_D_/*I*_G_ value (1.05) and BET surface area (69.67 m^2^ g^−1^), which imply that acetylene black has less defects than NOHC‐800. According to Equation (2), we have calculated the specific capacitive contribution in acetylene black. As shown in Figure S12c in the Supporting Information, the capacitive contribution of acetylene black is 51.3% at 0.8 mV s^−1^, much lower than that of NOHC‐800 (76.1%). Moreover, we have also compared the effect of defects on the capacitive behavior of NOHC‐800 with other reports in open literatures.^49^, ^50^, ^51^, ^52^, ^53^ For example, Lu et al. reported 3D amorphous carbon (3DAC) and common amorphous carbon (AC), which exhibit lower *I*_D_/*I*_G_ values (1.11 and 1.05 for 3DAC and AC, respectively) and BET surface areas (32.8 and 10.8 m^2^ g^−1^ for 3DAC and AC, respectively) than those of NOHC‐800.^49^ According to the Raman and BET surface areas, it can be inferred that 3DAC has fewer defects than NOHC‐800 but more defects than AC. Accordingly, 3DAC and AC show relatively lower capacitive contribution of 76% and 70% at 1.0 mV s^−1^, respectively, compared with that of NOHC‐800 (78.6%). Yao et al. fabricated microporous soft carbon nanosheets (SC‐NS) and the counterpart conventional soft carbon microrod (SC‐MR).^52^ The capacitance‐controlled capacity of SC‐NS (63.7%) is much higher than that of SC‐MR (40.6%) at 0.8 mV s^−1^ due to more defects in SC‐NS.

**Figure 6:**
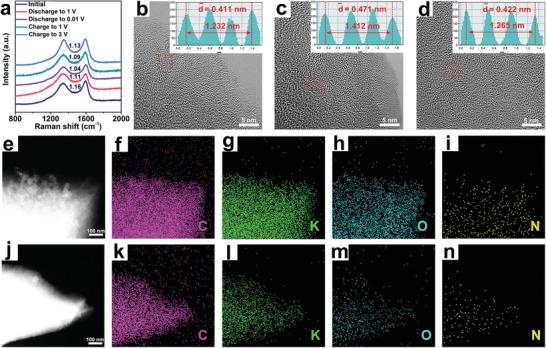
a) Ex situ Raman spectra of NOHC‐800 at various potassiation states. b–d) Ex situ HRTEM images of NOHC‐800 at initial, discharge, and charge states, respectively. e–i) EDS elemental mappings of C, K, O, N elements in NOHC‐800 at the discharge state. j–n) EDS elemental mappings of C, K, O, N elements in NOHC‐800 at the charge state.

To investigate the mechanism during the charge/discharge process, ex situ Raman analysis and HRTEM for the NOHC‐800 electrode in the initial, discharge, and charge states were carried out. Initially, the ex situ Raman analysis under different states are measured to elucidate the charge and discharge process (see **Figure** **6**a). During the discharge process, the value of *I*_D_/*I*_G_ decreases from 1.16 to 1.04, indicating the enhanced degree of graphitization. In the subsequent charge process, the value of *I*_D_/*I*_G_ increases from 1.09 to 1.13, demonstrating the diminished degree of graphitization.^13^, ^16^ As mentioned above (see Figure 2f,g), the interlayer spacing of NOHC‐800 in the initial state is 0.411 nm, and it reaches to 0.471 nm after full discharge (see Figure 6c), while recovers to 0.422 nm (see Figure 6d) after the full charge. Such a result proves the reversible intercalate/deintercalate of K^+^ in NOHC‐800 and its ultra‐stable structure. It is apparent that the flexible structure of NOHC‐800 can make it adapt to the enlarged interlayer spacing during the discharge process more easily. Consequently, NOHC‐800 shows high capacity, excellent rate performance, and long‐cycle stability. Furthermore, according to the energy dispersive spectrum (EDS) mapping (see Figure 6e–n) for the NOHC‐800 electrode after the discharge and charge process, C and K are dispersed uniformly with the stronger intensity of K after potassiation than that of depotassiation, further manifesting the successful intercalate/deintercalate of K^+^ in NOHC‐800. Note that the weak intensity of K after depotassiation is attributed to the residual K in SEI films. Additionally, the homogenous K elemental mapping implies that the generated SEI layer is uniform.^54^, ^55^

**Figure 7:**
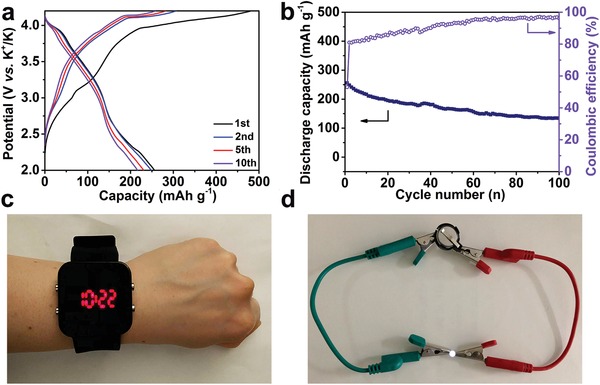
Electrochemical performances of the NOHC‐800// KPB potassium‐ion full‐cell. a) Galvanostatic charge/discharge profiles at 0.1 A g^−1^. b) Cycling performance and CE at 0.1 A g^−1^. c,d) The lighted light‐emitting diode (LED) watch and white LED bulb driven by the NOHC‐800//KPB potassium‐ion full‐cell.

To illuminate the practical performance of NOHC, we further assemble the coin‐type full‐cell with the NOHC‐800 and potassium Prussian blue (KPB) as the anode and cathode, respectively.^56^ It is worth noting that both materials are sustainable and cost‐effective. The characterizations of KPB are well consistent with previous work (see Figures S13 and S14, Supporting Information).^44^, ^56^ **Figure** **7**a shows the charge/discharge curves of the full‐cell at 0.1 A g^−1^ carried out in the potential range of 2.0–4.2 V. The full‐cell presents a high initial discharge capacity of 255.8 mAh g^−1^ (based on the mass of hard carbon anode), and it maintains 133.8 mAh g^−1^ after 100 cycle with a capacity retention of 52.3% (see Figure 7b), revealing good cyclability of the NOHC‐800//KPB full‐cell. Meanwhile, the full‐cell can lighten a wearable light‐emitting diode (LED) watch (see Figure 7c) and white LED bulb (see Figure 7d) after being fully charged, suggesting the promising applications prospect of NOHC‐800 as the anode for PIBs.

## 3 Conclusion

In summary, we have constructed a hybrid of NOHC using renewable piths of SSs as precursors through a simple, low‐cost, and large‐scale carbonization method. The NOHC shows super‐stable porous structure, expanded interlayer space, and N/O dual‐doping, thus providing more electrolyte/ions pathways, abundant effective active sites for K^+^ insertion/extraction, and low resistance for efficient electron transport. As a result, the NOHC electrode presents a high reversible capacity (304.6 mAh g^−1^ at 0.1 A g^−1^ after 100 cycles) and superior cycling stability (189.5 mAh g^−1^ at 1 A g^−1^ after 5000 cycles). Additionally, full‐cells ground on the NOHC and KPB exhibit relatively high capacity and stable cycling property, showing great potential for commercialization applications. This work may impel the development of low‐cost and sustainable carbon‐based materials for PIBs and other advanced energy storage devices.

## 4 Experimental Section

##### Synthesis of NOHCs

The NOHCs were prepared through a facile carbonization process using piths of SSs as precursor. First, the SSs were peeled the rinds and cut into small sections. The obtained products were washed with deionized water and ethanol several times and dried thoroughly. Then, the piths were ball‐milled for 10 min by using SPEX 8000 Mixer/Mill and carbonized in a tube furnace under Argon atmosphere at 600, 800, and 1000 °C for 2 h, respectively. The obtained hard carbon was immersed in 2 m HCl to remove metal impurities and then washed with deionized water to neutral. Finally, NOHCs were procured after drying and denoted as NOHC‐600, NOHC‐800, and NOHC‐1000, respectively.

##### Synthesis of KPB

The cathode material KPB was prepared through an easily precipitation method in an aqueous solution.^56^ Typically, 0.42 g of K_4_Fe(CN)_6_·3H_2_O was dissolved in 160 mL of deionized water to form solution A, and 0.32 g of FeCl_3_ was dissolved in 40 mL of deionized water to form solution B. Solution B was dropwise added into solution A under stirring and precipitation appeared immediately. The mixture was stirred for 2 h and aged for another 24 h at room temperature. The procured dark blue sediments were collected by centrifugation, washed with deionized water and ethanol several times, and dried at 80 °C in a vacuum oven for 24 h.

##### Materials Characterization

The morphology and microstructure of the samples were characterized by FESEM (JSM‐6700F, JEOL, 15 keV) and TEM (JEM‐2100F, JEOL, 200 keV). XRD was implemented on a D/max2500pc diffractometer using Cu‐K_α_ radiation. Raman spectra were gathered using a micro‐Raman spectrometer (Renishaw) with a 532 nm laser. XPS analysis was collected using an ESCALAB 250Xi system. The specific surface area and pore sizes were tested by nitrogen adsorption and desorption using a Micromeritics ASAP 2020 analyzer.

##### Electrochemical Measurements

The electrochemical measurements of the NOHCs anode materials were performed on coin‐type cells (2025 type), which were assembled in an argon‐filled glove box ([O_2_]<1 ppm, [H_2_O]<1 ppm) using as‐prepared NOHCs as electrode active materials, potassium foil as counter/reference electrode, and glass fiber (GF/C) as the separator. The electrolyte was 0.8 m KPF_6_ dissolved in a mixture of ethylene carbonate and diethyl carbonate at a volume ratio of 1:1. The working electrode was fabricated by mixing active materials, conductive material (Super P), and binder of sodium carboxymethylcellulose (Na‐CMC) with a weight ratio of 7:2:1 with deionized water as the solvent, which were uniformly pasted on Cu foil and dried in vacuum at 60 °C for 12 h. The mass of the active material in the working electrode was 0.6–1.0 mg. A LAND‐CT2011A battery testing system was used to measure the galvanostatic charge/discharge curves at an ambient temperature in a potential range of 0.01–3.0 V (vs K^+^/K). CV measurements were recorded on an IVIUM electrochemical workstation at a scan rate of 0.1 mV s^−1^ in the range of 0.01–3.0 V (vs K^+^/K). EIS measurements were implemented with an amplitude of 10 mV over the frequency range of 100 kHz to 10 mHz. The full‐cell was fabricated using KPB as cathode and NOHCs as anode, with the same electrolyte and separator. The cathode electrodes were fabricated by mixing 60 wt% KPB, 30 wt% conductive agent (Super P), and 10 wt% binder (polyvinylidene fluoride) using *N*‐methyl‐2‐pyrrolidone as a solvent, and then pasted on Al foil which was dried in vacuum at 110 °C for 12 h. The cathode‐to‐anode mass loading ratio was 5:1 to assure the optimized property of the as‐prepared full‐cell. Galvanostatic charge/discharge tests of the full‐cell were carried out in a potential range of 2.0–4.2 V at 0.1 A g^−1^ (based on the active material mass of the anode).

## Conflict of Interest

The authors declare no conflict of interest.

## Supporting information

Supporting Information

## References

[1] M. Armand , J. M. Tarascon , Nature 2008, 451, 652. 18256660 10.1038/451652a

[2] C. C. Yang , W. T. Jing , C. Li , Q. Jiang , J. Mater. Chem. A 2018, 6, 3877.

[3] D. Wang , W. W. Zhou , R. Zhang , J. J. Zeng , Y. Du , S. Qi , C. X. Cong , C. Y. Ding , X. X. Huang , G. W. Wen , T. Yu , Adv. Mater. 2018, 30, 1803569. 10.1002/adma.201803569 30252169

[4] Z. H. Zhang , B. B. Chen , H. M. Xu , Z. L. Cui , S. M. Dong , A. B. Du , J. Ma , Q. F. Wang , X. H. Zhou , G. L. Cui , Adv. Funct. Mater. 2018, 28, 1701718.

[5] D. Wang , X. W. Gao , Y. H. Chen , L. Y. Jin , C. Kuss , P. G. Bruce , Nat. Mater. 2018, 17, 16. 29180779 10.1038/nmat5036

[6] D. Y. Wang , C. Y. Wei , M. C. Lin , C. J. Pan , H. L. Chou , H. A. Chen , M. Gong , Y. P. Wu , C. Z. Yuan , M. Angell , Y. J. Hsieh , Y. H. Chen , C. Y. Wen , C. W. Chen , B. J. Hwang , C. C. Chen , H. J. Dai , Nat. Commun. 2017, 8, 14283. 28194027 10.1038/ncomms14283 PMC5316828

[7] T. H. Cai , L. M. Zhao , H. Y. Hu , T. G. Li , X. C. Li , S. Guo , Y. P. Li , Q. Z. Xue , W. Xing , Z. F. Yan , L. Z. Wang , Energy Environ. Sci. 2018, 11, 2341.

[8] G. Z. Li , B. Huang , Z. F. Pan , X. Y. Su , Z. P. Shao , L. An , Energy Environ. Sci. 2019, 12, 2030.

[9] M. B. Yahia , J. Vergnet , M. Saubanère , M. L. Doublet , Nat. Mater. 2019, 18, 496. 30886397 10.1038/s41563-019-0318-3

[10] C. C. Yang , D. M. Zhang , L. Du , Q. Jiang , J. Mater. Chem. A 2018, 6, 12663.

[11] W. T. Jing , Y. Zhang , Y. Gu , Y. F. Zhu , C. C. Yang , Q. Jiang , Matter 2019, 1, 720.

[12] Y. H. Zhu , Q. Zhang , X. Yang , E. Y. Zhao , T. Sun , X. B. Zhang , S. Wang , X. Q. Yu , J. M. Yan , Q. Jiang , Chem 2019, 5, 168.

[13] W. X. Yang , J. H. Zhou , S. Wang , W. Y. Zhang , Z. C. Wang , F. Lv , K. Wang , Q. Sun , S. J. Guo , Energy Environ. Sci. 2019, 12, 1605.

[14] W. Wang , B. Jiang , C. Qian , F. Lv , J. R. Feng , J. H. Zhou , K. Wang , C. Yang , Y. Yang , S. J. Guo , Adv. Mater. 2018, 30, 1801812. 10.1002/adma.201801812 29894007

[15] W. Wang , J. H. Zhou , Z. P. Wang , L. Y. Zhao , P. H. Li , Y. Yang , C. Yang , H. X. Huang , S. J. Guo , Adv. Energy Mater. 2018, 8, 1701648.

[16] M. Chen , W. Wang , X. Liang , S. Gong , J. Liu , Q. Wang , S. J. Guo , H. Yang , Adv. Energy Mater. 2018, 8, 1800171.

[17] J. F. Ruan , Y. H. Zhao , S. N. Luo , T. Yuan , J. H. Yang , D. L. Sun , S. Y. Zheng , Energy Storage Mater. 2019, 23, 46.

[18] W. C. Zhang , Y. J. Liu , Z. P. Guo , Sci. Adv. 2019, 5, eaav7412. 31093528 10.1126/sciadv.aav7412 PMC6510555

[19] Y. J. Liu , Z. X. Tai , J. Zhang , W. K. Pang , Q. Zhang , H. F. Feng , K. Konstantinov , Z. P. Guo , H. K. Liu , Nat. Commun. 2018, 9, 3645. 30194304 10.1038/s41467-018-05786-1 PMC6128879

[20] Y. Wu , H. B. Huang , Y. Z. Feng , Z. S. Wu , Y. Yu , Adv. Mater. 2019, 31, 1901414. 10.1002/adma.201901414 31456280

[21] Q. Zhang , C. Didier , W. K. Pang , Y. J. Liu , Z. J. Wang , S. Li , V. K. Peterson , J. F. Mao , Z. P. Guo , Adv. Energy Mater. 2019, 9, 1900568.

[22] D. S. Bin , X. J. Lin , Y. G. Sun , Y. S. Xu , K. Zhang , A. M. Cao , L. J. Wan , J. Am. Chem. Soc. 2018, 140, 7127. 29771119 10.1021/jacs.8b02178

[23] C. Zeng , F. X. Xie , X. F. Yang , M. Jaroniec , L. Zhang , S. Z. Qiao , Angew. Chem., Int. Ed. 2018, 57, 8540. 10.1002/anie.201803511 29722102

[24] J. Q. Huang , X. Y. Lin , H. Tan , B. Zhang , Adv. Energy Mater. 2018, 8, 1703496.

[25] J. Zheng , Y. Yang , X. L. Fan , G. B. Ji , X. Ji , H. Y. Wang , S. Y. Hou , M. R. Zachariah , C. S. Wang , Energy Environ. Sci. 2019, 12, 615.

[26] Y. L. An , Y. Tian , L. J. Ci , S. L. Xiong , J. K. Feng , Y. T. Qian , ACS Nano 2018, 12, 12932. 30481455 10.1021/acsnano.8b08740

[27] H. X. Yu , X. Cheng , M. T. Xia , T. T. Liu , W. Q. Ye , R. T. Zheng , N. B. Long , M. Shui , J. Shu , Energy Storage Mater. 2019, 22, 154.

[28] H. N. He , D. Huang , Y. G. Tang , Q. Wang , X. B. Ji , H. Y. Wang , Z. P. Guo , Nano Energy 2019, 57, 728.

[29] W. C. Zhang , J. F. Mao , S. Li , Z. X. Chen , Z. P. Guo , J. Am. Chem. Soc. 2017, 139, 3316. 28211269 10.1021/jacs.6b12185

[30] H. Gao , T. F. Zhou , Y. Zheng , Q. Zhang , Y. Q. Liu , J. Chen , H. K. Liu , Z. P. Guo , Adv. Funct. Mater. 2017, 27, 1702634.

[31] B. Bakeer , I. Taha , H. E. Mously , S. A. Shehata , Ain Shams Eng. J. 2013, 4, 265.

[32] Y. M. Li , Y. S. Hu , M. M. Titrici , L. Q. Chen , X. J. Huang , Adv. Energy Mater. 2016, 6, 1600659.

[33] J. Ding , H. L. Wang , Z. Li , K. Cui , D. Karpuzov , X. H. Tan , A. Kohandehghan , D. Mitlin , Energy Environ. Sci. 2015, 8, 941.

[34] P. Liu , Y. M. Li , Y. S. Hu , H. Li , L. Q. Chen , X. J. Huang , J. Mater. Chem. A 2016, 4, 13046.

[35] X. Zhou , P. L. Wang , Y. G. Zhang , L. L. Wang , L. T. Zhang , L. Zhang , L. Xu , L. Liu , J. Mater. Chem. A 2017, 5, 12958.

[36] R. B. Lockman , Commun. Soil Sci. Plant Anal. 1972, 3, 295.

[37] Z. L. Jian , W. Luo , X. L. Ji , J. Am. Chem. Soc. 2015, 137, 11566. 26333059 10.1021/jacs.5b06809

[38] R. Hao , H. Lan , C. W. Kuang , H. Wang , L. Guo , Carbon 2018, 128, 224.

[39] Y. H. Xie , Y. Chen , L. Liu , P. Tao , M. P. Fan , N. Xu , X. W. Shen , C. L. Yan , Adv. Mater. 2017, 29, 1702268. 10.1002/adma.201702268 28714252

[40] J. L. Yang , Z. C. Ju , Y. Jiang , Z. Xing , B. J. Xi , J. K. Feng , S. L. Xiong , Adv. Mater. 2018, 30, 1700104. 10.1002/adma.201700104 29215156

[41] C. Q. Yuan , X. H. Liu , M. Y. Jia , Z. X. Luo , J. N. Yao , J. Mater. Chem. A 2015, 3, 3409.

[42] F. C. Zheng , Y. Yang , Q. W. Chen , Nat. Commun. 2014, 5, 5261. 25374050 10.1038/ncomms6261

[43] Y. S. Wang , Z. P. Wang , Y. J. Chen , H. Zhang , M. Yousaf , H. S. Wu , M. C. Zou , A. Y. Cao , R. P. S. Han , Adv. Mater. 2018, 30, 1802074. 10.1002/adma.201802074 29952034

[44] Y. Xu , C. L. Zhang , M. Zhou , Q. Fu , C. X. Zhao , M. H. Wu , Y. Lei , Nat. Commun. 2018, 9, 1720. 29712922 10.1038/s41467-018-04190-z PMC5928078

[45] L. F. Xiao , Y. L. Cao , W. A. Henderson , M. L. Sushko , Y. Y. Shao , J. Xiao , W. Wang , M. H. Engelhard , Z. M. Nie , J. Liu , Nano Energy 2016, 19, 279.

[46] V. Augustyn , J. Come , M. A. Lowe , J. W. Kim , P. L. Taberna , S. H. Tolbert , H. D. Abruña , P. Simon , B. Dunn , Nat. Mater. 2013, 12, 518. 23584143 10.1038/nmat3601

[47] D. W. Su , A. McDonagh , S.‐Z. Qiao , G. X. Wang , Adv. Mater. 2017, 29, 1604007.

[48] Y. J. Fang , X. Y. Yu , X. W. Lou , Adv. Mater. 2018, 30, 1706668. 10.1002/adma.201706668 29633418

[49] P. Lu , Y. Sun , H. F. Xiang , X. Liang , Y. Yu , Adv. Energy Mater. 2018, 8, 1702434.

[50] G. Z. Fang , C. Y. Zhu , M. H. Chen , J. Zhou , B. Y. Tang , X. X. Cao , X. S. Zheng , A. Q. Pan , S. Q. Liang , Adv. Funct. Mater. 2019, 29, 1808375.

[51] T. Xiong , Z. G. Yu , H. J. Wu , Y. H. Du , Q. D. Xie , J. S. Chen , Y. W. Zhang , S. J. Pennycook , W. S. V. Lee , J. M. Xue , Adv. Energy Mater. 2019, 9, 1803815.

[52] X. H. Yao , Y. J. Ke , W. H. Ren , X. P. Wang , F. Y. Xiong , W. Yang , M. S. Qin , Q. Li , L. Q. Mai , Adv. Energy Mater. 2019, 9, 1803260.

[53] Y. Xu , F. Bahmani , M. Zhou , Y. L. Li , C. L. Zhang , F. Liang , S. H. Kazemi , U. Kaiser , G. W. Meng , Y. Lei , Nanoscale Horiz. 2019, 4, 202. 10.1039/c8nh00305j 32254157

[54] D. M. Zhang , J. H. Jia , C. C. Yang , Q. Jiang , Energy Storage Mater. 2020, 24, 439.

[55] S. H. Choi , Y. N. Ko , J. K. Lee , Y. C. Kang , Adv. Funct. Mater. 2015, 25, 1780.

[56] C. L. Zhang , Y. Xu , M. Zhou , L. Y. Liang , H. S. Dong , M. H. Wu , Y. Yang , Y. Lei , Adv. Funct. Mater. 2017, 27, 1604307.

