# Supplementary material for: N/O Dual‐Doped Environment‐Friendly Hard Carbon as Advanced Anode for Potassium‐Ion Batteries
Source: Adv Sci (Weinh). 2020 Jan 9;7(5):1902547. doi: 10.1002/advs.201902547 (PMC7055548; doi:10.1002/advs.201902547)
Supplement: Supplementary file 1 — Supporting Information [file ADVS-7-1902547-s001.pdf]

Copyright WILEY-VCH Verlag GmbH & Co. KGaA, 69469 Weinheim, Germany, 2020.

ADVANCED SCIENCE Open Access

Supporting Information

for *Adv. Sci.,* DOI: 10.1002/advs.201902547

N/O Dual-Doped Environment-Friendly Hard Carbon as Advanced Anode for Potassium-Ion Batteries

Rong Chao Cui, Bo Xu, Hou Ji Dong, Chun Cheng Yang, and Qing Jiang**

Copyright WILEY-VCH Verlag GmbH & Co. KGaA, 69469 Weinheim, Germany, 2018.

Supporting Information

N/O Dual-Doped Environment-Friendly Hard Carbon as Advanced Anode for Potassium-Ion Batteries

Rong Chao Cui, Bo Xu, Hou Ji Dong, Chun Cheng Yang, and Qing Jiang**

R. C. Cui, B. Xu, H. J. Dong, Prof. C. C. Yang, Prof. Q. Jiang
Key Laboratory of Automobile Materials (Jilin University), Ministry of Education, and School of Materials Science and Engineering, Jilin University
Changchun 130022, China
E-mails: ccyang@jlu.edu.cn (C. C. Yang); jiangq@jlu.edu.cn (Q. Jiang)

Supplementary Figures

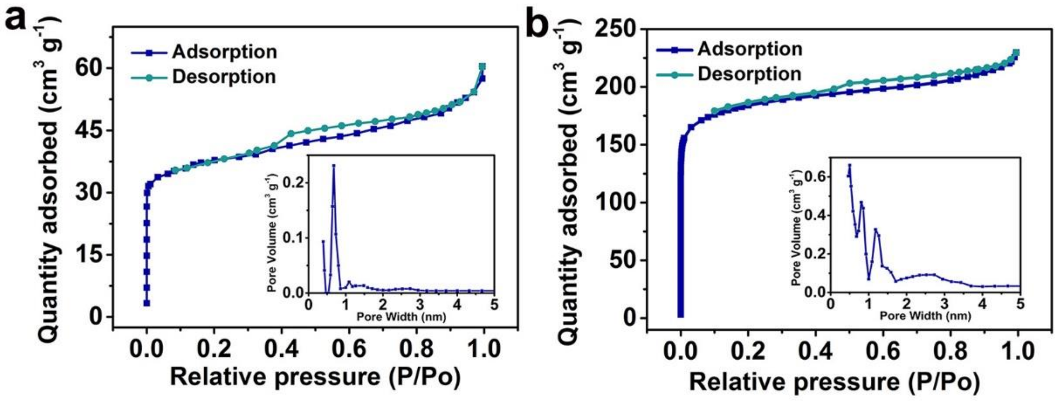

Figure S1. N_2 adsorption-desorption isotherms of a) NOHC-600 and b) NOHC-1000. The insert shows the pore size distribution of the adsorption branch obtained by the Density Functional Theory (DFT) method.

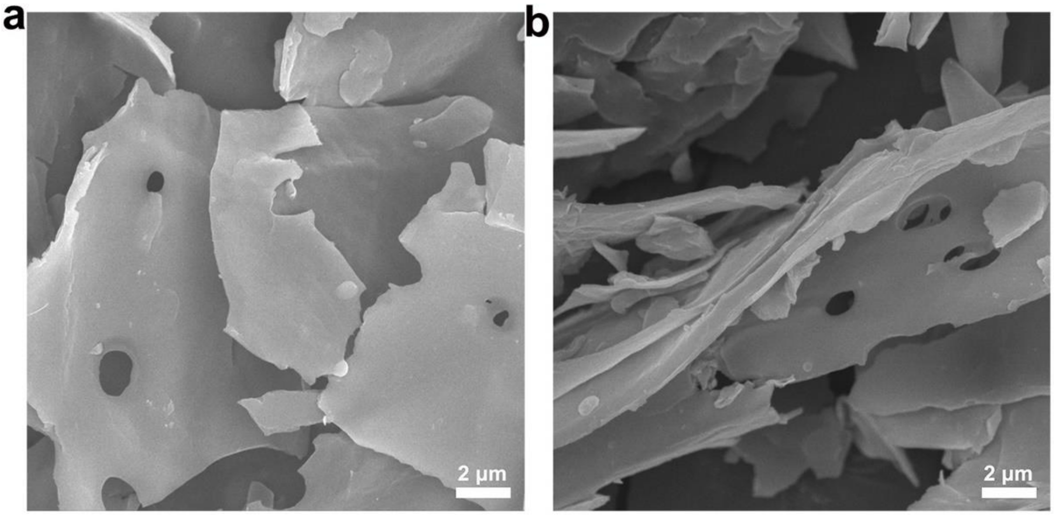

Figure S2. FESEM images of a) NOHC-600 and b) NOHC-1000.

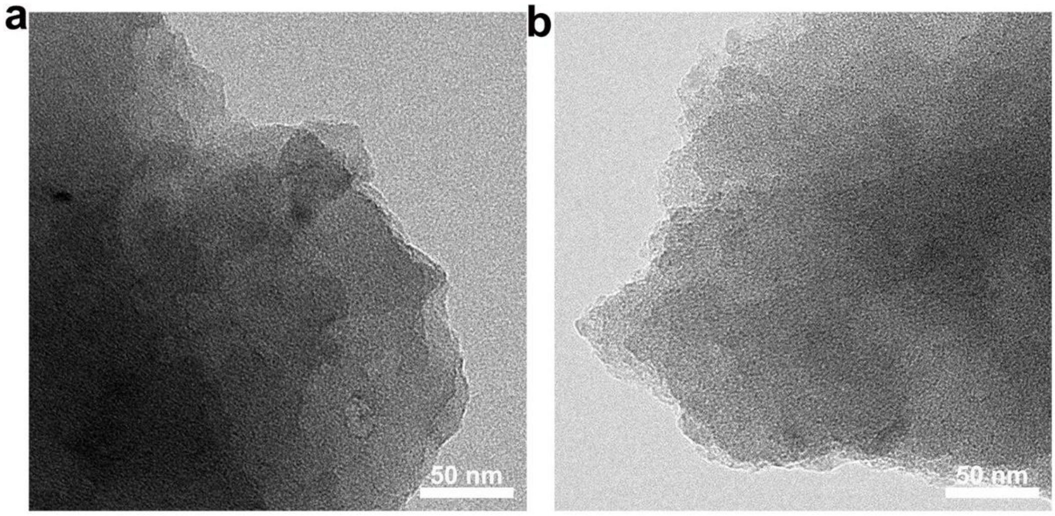

Figure S3. TEM images of a) NOHC-600 and b) NOHC-1000.

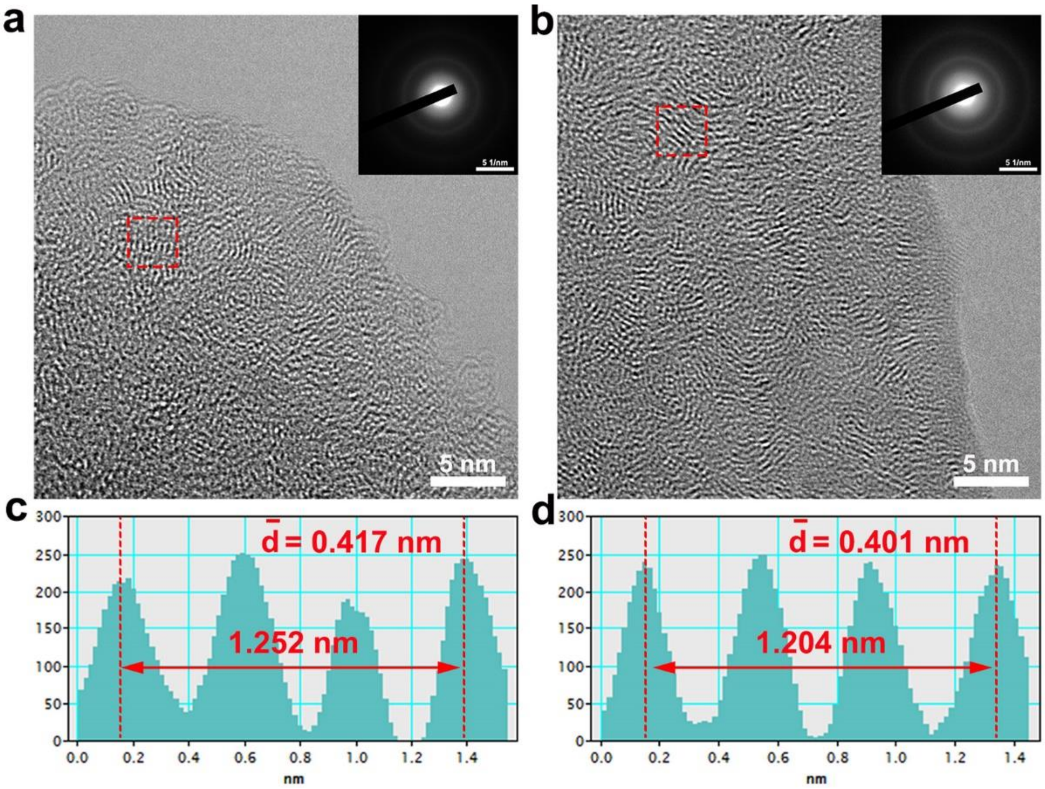

Figure S4. HRTEM images of a) NOHC-600 and b) NOHC-1000. Line profiles of c) NOHC-600 and d) NOHC-1000 acquired from the framed area in a) and b), respectively.

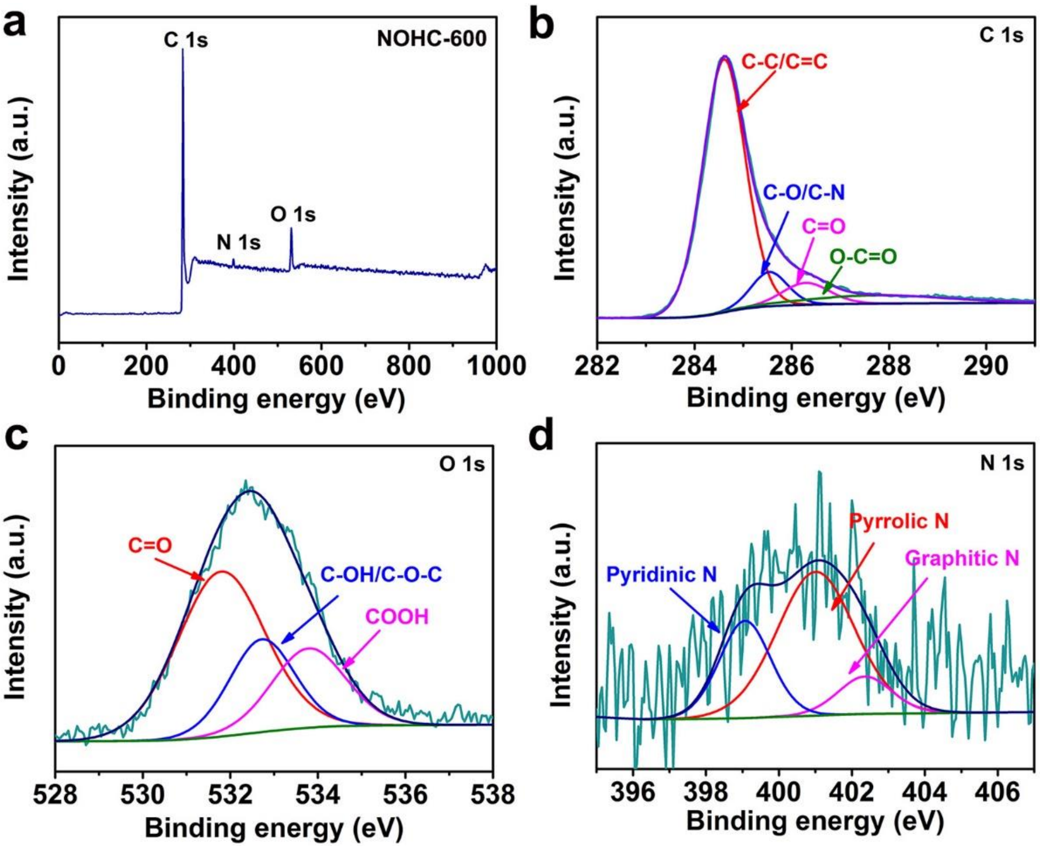

Figure S5. XPS survey spectra of NOHC-600. a) The survey spectrum. b-d) are high-resolution XPS spectra of C 1s, O 1s and N 1s, respectively.

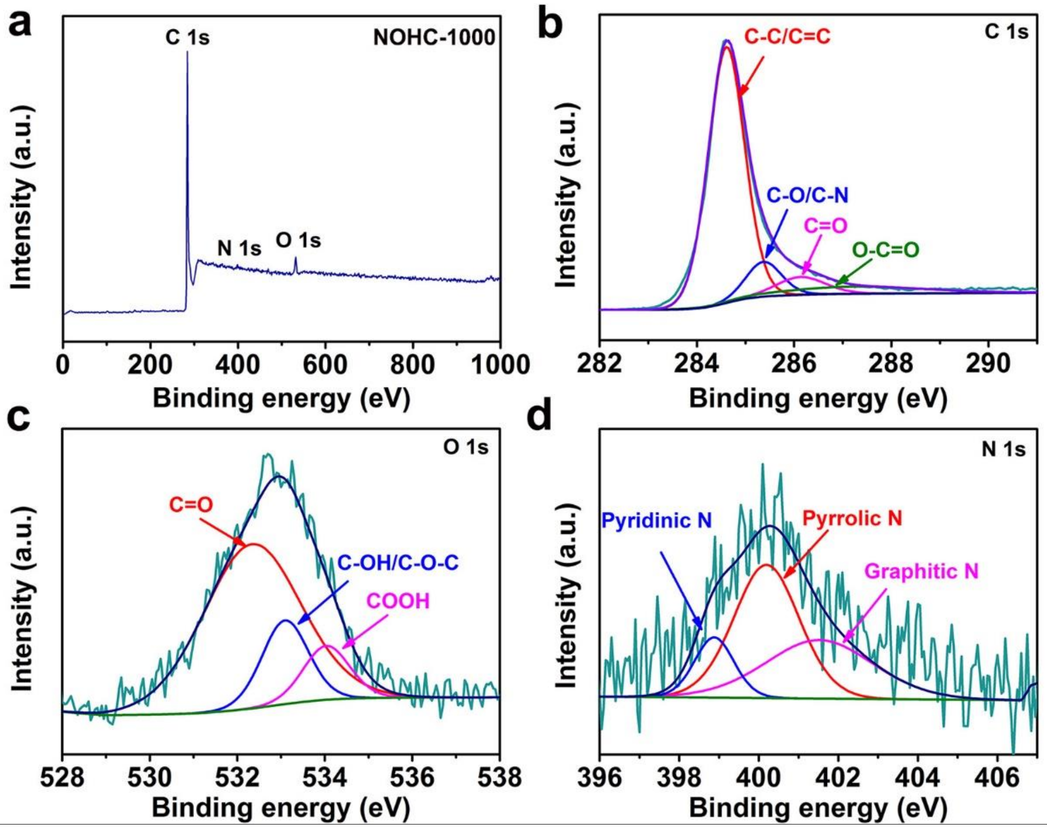

Figure S6. XPS survey spectra of NOHC-1000. a) The survey spectrum. b-d) are high-resolution XPS spectra of C 1s, O 1s and N 1s, respectively.

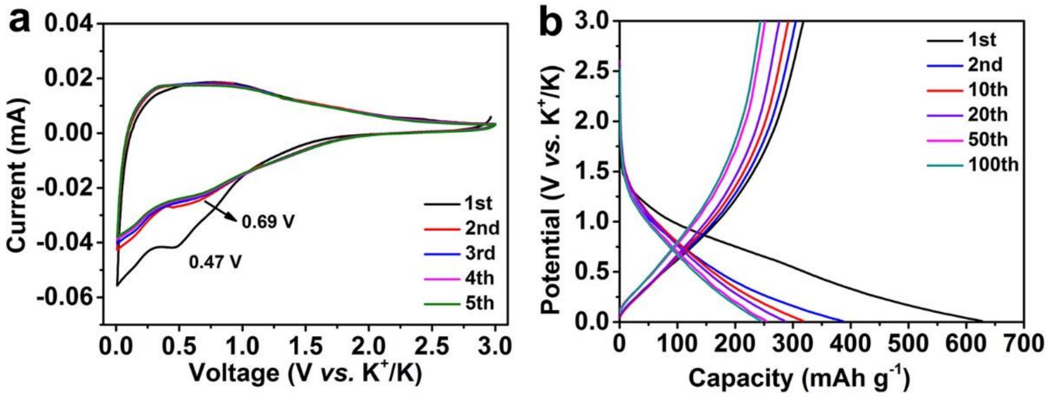

Figure S7. a) CV and b) galvanostatic charge/discharge curves of NOHC-600.

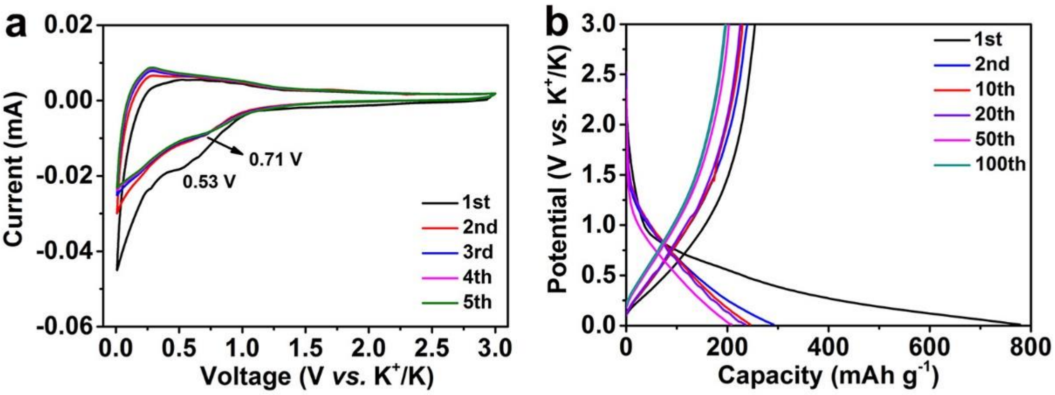

Figure S8. a) CV and b) galvanostatic charge/discharge curves of NOHC-1000.

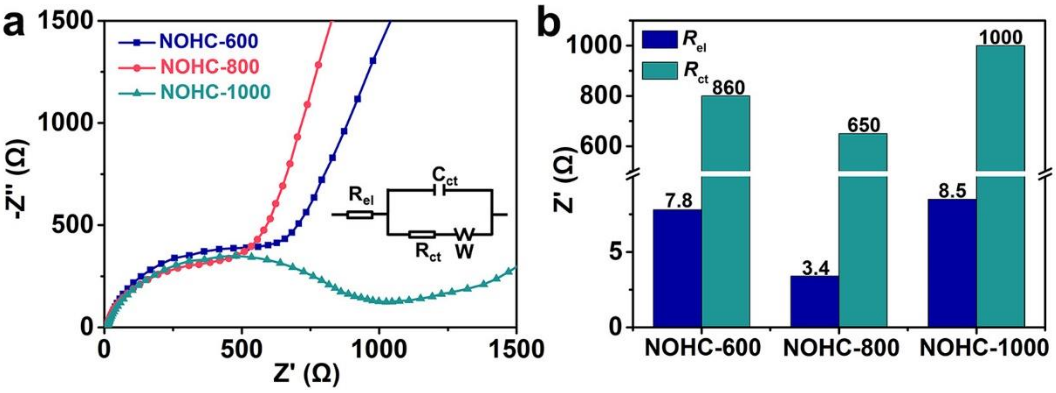

Figure S9. a) EIS spectra of NOHC-600, NOHC-800 and NOHC-1000, where the inset shows the equivalent circuit diagram. b) The electrolyte resistance (R_{el}) and charge transfer resistance (R_{ct}) values of NOHC-600, NOHC-800 and NOHC-1000.

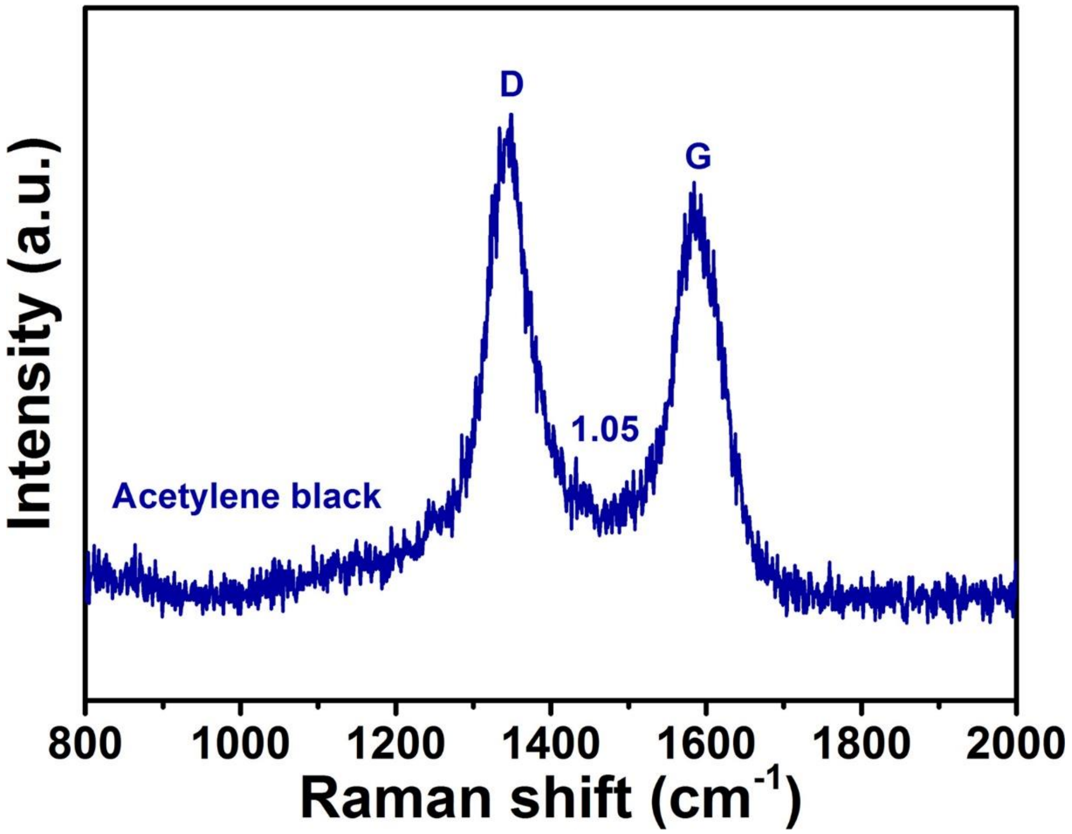

Figure S10. Raman spectrum of acetylene black.

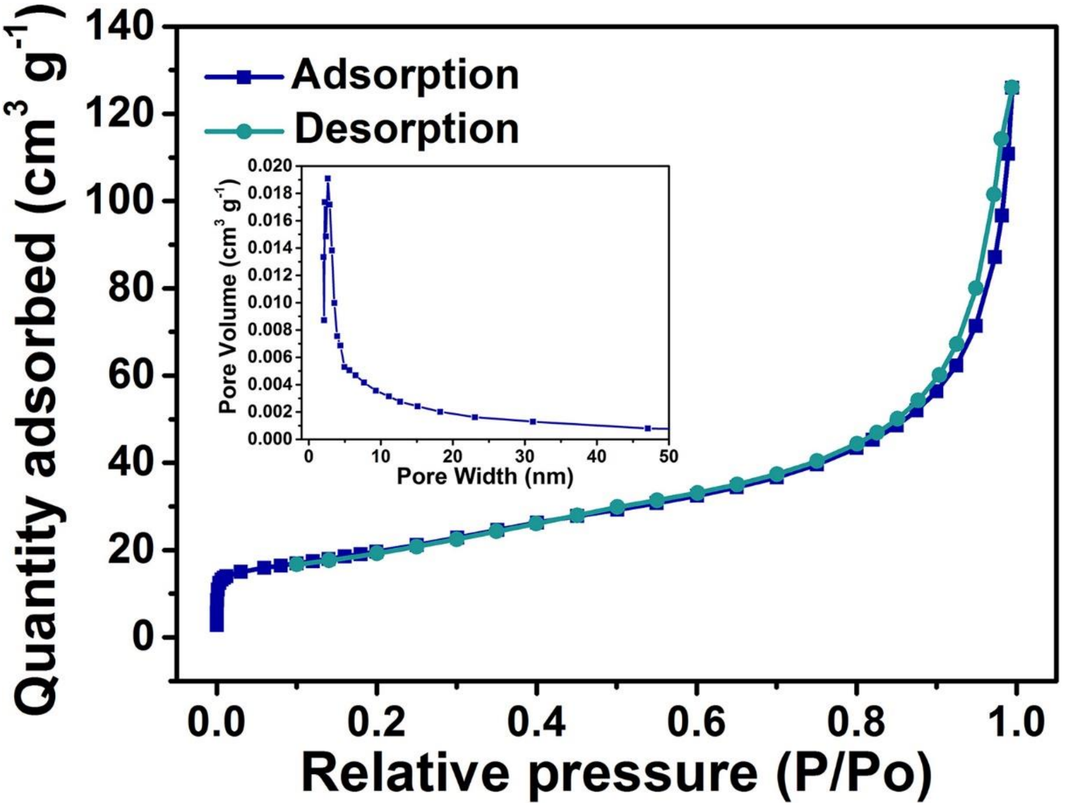

Figure S11. BET surface area of acetylene black.

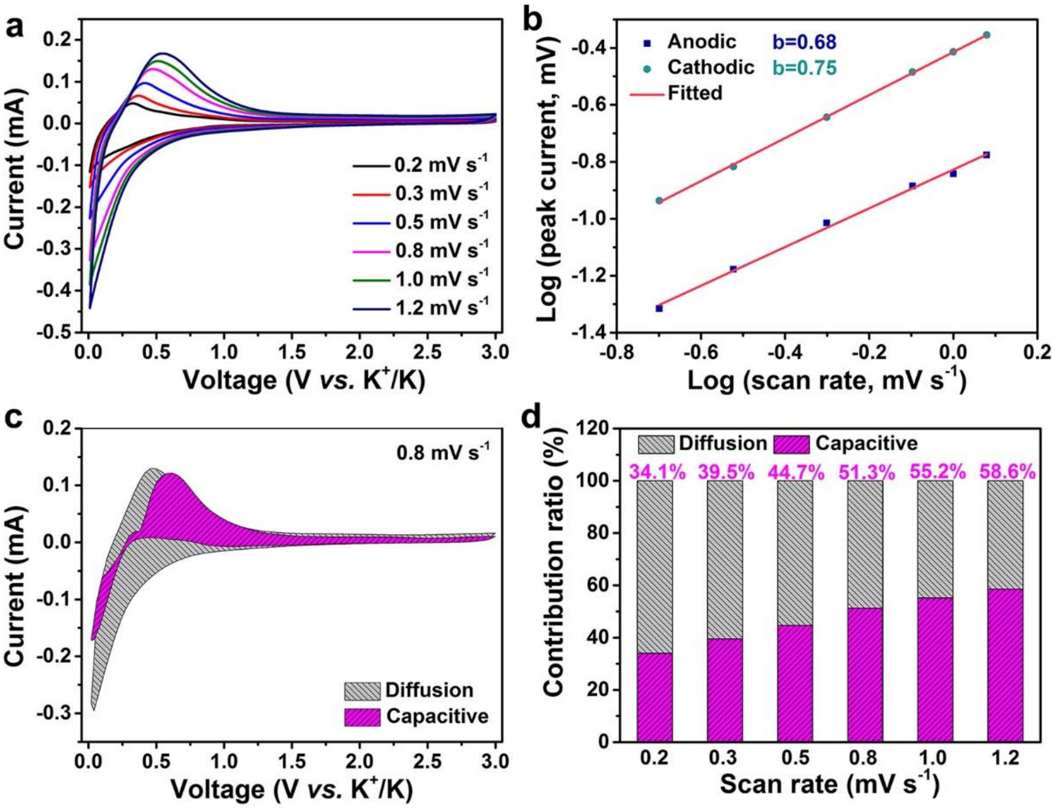

Figure S12. Quantitative analysis of potassium-ion storage in acetylene black. a) Cyclic voltammetry curves at various scan rates from 0.2 to 1.2 mV s^{-1}. b) The measurement of b-value. The b-values of anodic and cathodic are 0.68 and 0.75, respectively. c) Contribution of the capacitive and diffusion process at a scan rate of 0.8 mV s^{-1}. d) Contribution ratios of the capacitive process at different scan rates.

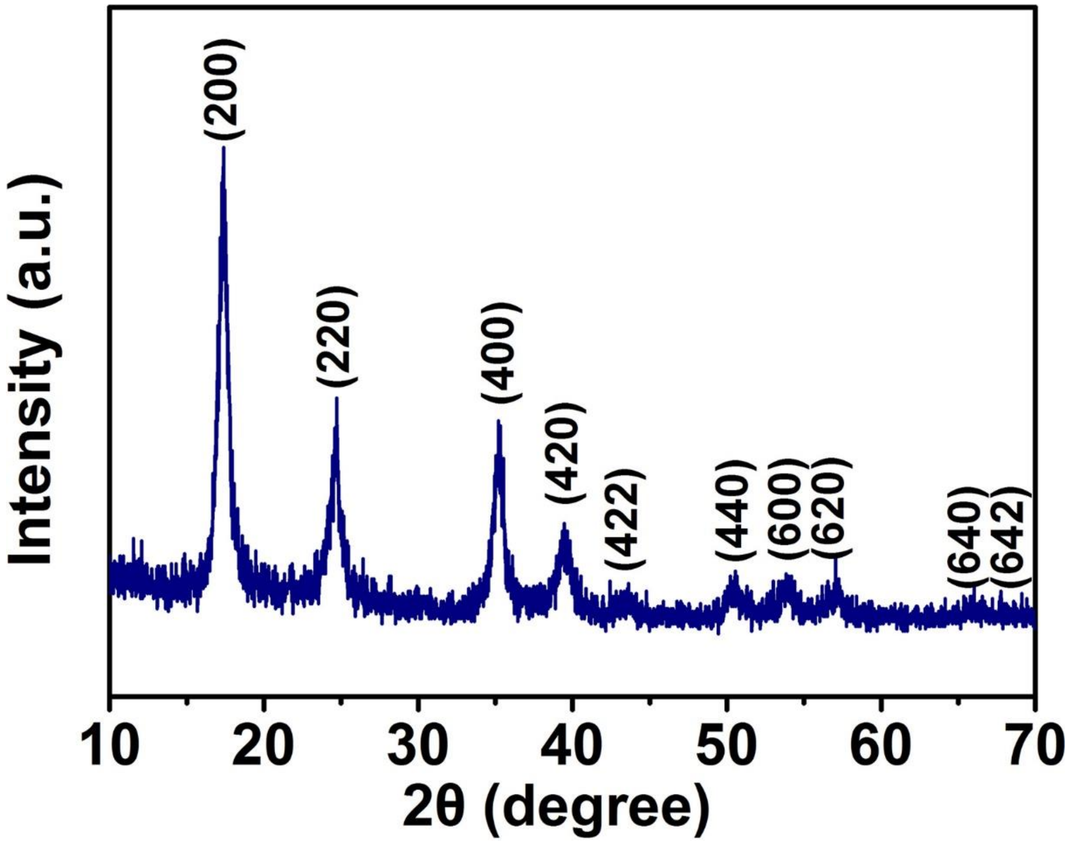

Figure S13. XRD pattern of potassium prussian blue (KPB).

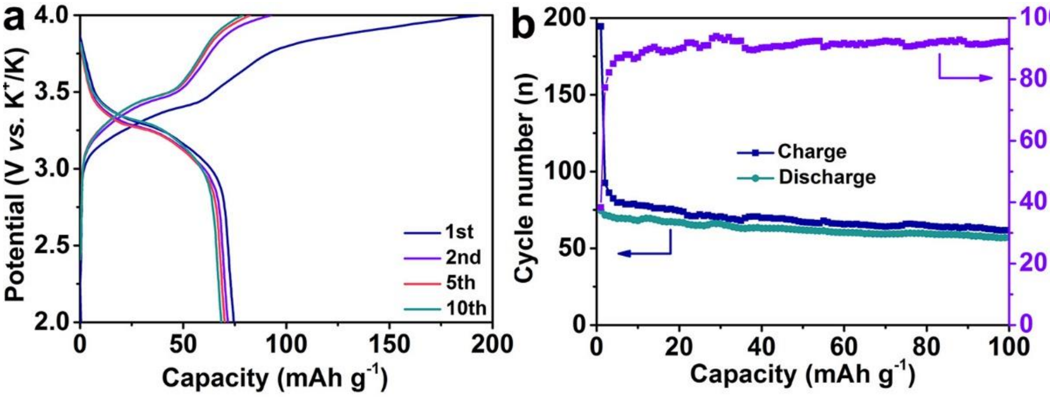

Figure S14. Electrochemical performance of KPB. a) Galvanostatic charge/discharge profiles at 0.1 A g^{-1} in a potential range of 0.01-3.0 V. b) Cycling performance at 0.1 A g^{-1}.

Supplementary Tables

Table S1. Comparisons of the cycling performance of NOHC-800 electrode with other carbon-based anode materials in PIBs reported in open literature.

Sample	Cycling performance	Ref.
NOHC-800	189.5 mAh g^{-1} (5000 cycles, 1 A g^{-1})	This work
N doped-porous carbon	152 mAh g^{-1} (3000 cycles, 1 A g^{-1})	Ref. [35] of the txt
Amorphous ordered mesoporous carbon	146.5 mAh g^{-1} (1000 cycles, 1 A g^{-1})	Ref. [15] of the txt
S/O dual-doped porous carbon microspheres	108.4 mAh g^{-1} (2000 cycles, 1 A g^{-1})	Ref. [16] of the txt
P/N dual-doped porous carbon	270.4 mAh g^{-1} (1000 cycles, 1 A g^{-1})	Ref. [24] of the txt
N/O dual-doped hard carbon	124.8 mAh g^{-1} (1100 cycles, 1.05 A g^{-1})	Ref. [36] of the txt
N doped carbon nanosheets	151 mAh g^{-1} (1000 cycles, 1 A g^{-1})	[1]
N doped bamboo-like carbon nanotubes	204 mAh g^{-1} (1000 cycles, 0.5 A g^{-1})	[2]
Highly N doped carbon nanofibers	146 mAh g^{-1} (4000 cycles, 2 A g^{-1})	Ref. [40] of the txt
Necklace-like N doped hollow carbon	161.3 mAh g^{-1} (1600 cycles, 1 A g^{-1})	Ref. [13] of the txt
N rich hard carbon	180 mAh g^{-1} (4000 cycles, 0.5 A g^{-1})	[3]
N/O dual-doped carbon network	160 mAh g^{-1} (4000 cycles, 1 A g^{-1})	Ref. [17] of the txt

References

[1] L. Liu, Y. Chen, Y. H. Xie, P. Tao, Q. Y. Li, C. L. Yan, *Adv. Funct. Mater.* **2018**, *28*, 1801989.

[2] Y. Liu, C. Yang, Q. Pan, Y. Li, G. Wang, X. Ou, F. Zheng, X. Xiong, M. Liu, Q. Zhang, *J. Mater. Chem. A* **2018**, *6*, 15162.

[3] C. J Chen, Z. G. Wang, B. Zhang, L. Miao, J. Cai, L. F. Peng, Y. Y. Huang, J. J. Jiang, Y. H. Huang, L. N. Zhang, J. Xie, *Energy Storage Mater.* **2017**, *8*, 161.
